# Multiple histone modifications in euchromatin promote heterochromatin formation by redundant mechanisms in *Saccharomyces cerevisiae*

**DOI:** 10.1186/1471-2199-10-76

**Published:** 2009-07-28

**Authors:** Kitty F Verzijlbergen, Alex W Faber, Iris JE Stulemeijer, Fred van Leeuwen

**Affiliations:** 1 Fred van Leeuwen, Division of Gene Regulation B4, Netherlands Cancer Institute, The Netherlands

## Abstract

**Background:**

Methylation of lysine 79 on histone H3 by Dot1 is required for maintenance of heterochromatin structure in yeast and humans. However, this histone modification occurs predominantly in euchromatin. Thus, Dot1 affects silencing by indirect mechanisms and does not act by the recruitment model commonly proposed for histone modifications. To better understand the role of H3K79 methylation gene silencing, we investigated the silencing function of Dot1 by genetic suppressor and enhancer analysis and examined the relationship between Dot1 and other global euchromatic histone modifiers.

**Result:**

We determined that loss of H3K79 methylation results in a partial silencing defect that could be bypassed by conditions that promote targeting of Sir proteins to heterochromatin. Furthermore, the silencing defect in strains lacking Dot1 was dependent on methylation of H3K4 by Set1 and histone acetylation by Gcn5, Elp3, and Sas2 in euchromatin. Our study shows that multiple histone modifications associated with euchromatin positively modulate the function of heterochromatin by distinct mechanisms. Genetic interactions between Set1 and Set2 suggested that the H3K36 methyltransferase Set2, unlike most other euchromatic modifiers, negatively affects gene silencing.

**Conclusion:**

Our genetic dissection of Dot1's role in silencing in budding yeast showed that heterochromatin formation is modulated by multiple euchromatic histone modifiers that act by non-overlapping mechanisms. We discuss how euchromatic histone modifiers can make negative as well as positive contributions to gene silencing by competing with heterochromatin proteins within heterochromatin, within euchromatin, and at the boundary between euchromatin and heterochromatin.

## Background

Post-translational modifications of histone proteins influence DNA transactions such as transcription, repair, recombination, and chromosome segregation. Many histone modifications affect local chromatin structure and function by recruitment of effector proteins that specifically recognize a modified state of a given residue [reviewed in [1-4]]. However, several histone modifications seem to act by alternative mechanisms. One such example is methylation of lysine 79 of histone H3 (H3K79) by Dot1. H3K79 methylation is required for heterochromatin formation in yeast and humans [5-10]. Paradoxically, methylation of H3K79 is low or absent from heterochromatic regions and is abundant in euchromatic regions of the genome [5,7,11-14]. Furthermore, methylation of H3K79, which causes small local changes of the nucleosome surface [15], negatively affects binding of the heterochromatin protein Sir3 in yeast [16-18]. Therefore, this histone modification most likely affects heterochromatin structure by mechanisms other than direct recruitment of repressive factors. We previously proposed that H3K79 methylation in yeast might act as an anti-binding signal to prevent non-specific binding of silencing proteins in euchromatin, thereby leading to efficient targeting of the limiting silencing proteins to the unmethylated heterochromatic regions of the genome [5,19].

Heterochromatin in yeast, often referred to as silent chromatin, is found at telomeres, the silent mating type loci (*HMLα *and *HMR***a**) and the ribosomal DNA repeats. At telomeres and *HM *loci, DNA elements called silencers recruit the Sir2/3/4 complex, which subsequently spreads along the chromosome to form a silent or heterochromatic domain [reviewed in [20]]. Besides H3K79 methylation, methylation of H3K4 and H3K36, histone acetylation, and deposition of the histone variant Htz1 (H2A.Z) in euchromatin have been shown to affect heterochromatin formation in yeast [reviewed in [20]]. Some euchromatic modifications have been suggested to act by (indirect) global effects, whereas others have been suggested to primarily act (directly) at the boundary between euchromatin and heterochromatin to prevent excessive spreading of the Sir2/3/4 complex. For example, loss of the histone variant Htz1, the H3K36 methyltransferase Set2, or the histone acetyltransferase Sas2 leads to loss of heterochromatin boundaries and excessive spreading at yeast telomeres [21-24], whereas in cells lacking Dot1 or the histone H3K4 methyltransferase Set1, Sir proteins become redistributed throughout the genome [5,25,26]. Methylation of H3K4 in euchromatin negatively affects binding of the C-terminus of Sir3, which led to the suggestion that Set1 enhances silencing by a mechanism similar to that of Dot1 [27].

The molecular mechanisms responsible for the different silencing functions of many of the euchromatic histone marks are still largely unknown. Here we used genetic suppressor and enhancer analysis to investigate the role of Dot1 in heterochromatin formation and its connection with several other global histone modifiers (see Table 1). We found that the silencing defect in strains lacking Dot1 was partial and could be suppressed by conditions that promote targeting of the Sir complex to telomeres. These results are in agreement with the proposed function of Dot1 in preventing non-specific binding to euchromatin. We show that Dot1 functions in parallel with the histone methyltransferase Set1 and histone acetyltransferases, suggesting that multiple euchromatic histone modifications promote silencing by non-overlapping mechanisms.

**Table 1:** Chromatin modifiers analyzed in this study

**Protein**	Alias*	**Enzymatic Complex**	**Target sites**
Dot1	KMT4	-	H3K79me1,2,3

Set1	KMT2	Compass	H3K4me1,2,3, Dam1

Set2	KMT3	-	H3K36me1,2,3

Gcn5	KAT2	SAGA, SALSA, SLIK, HatB3.1	H3K9,14,18,23,36; H2B, Htz1K14, Rsc4

Elp3	KAT9	Elongator	H3

Sas2	KAT8	SAS	H4K16

Sas3	KAT6	NuA3	H3K14,23

Eaf1		NuA4 (KAT)	H4; H2A; Htz1

Rpd3		Rpd3L, Rpd3S (HDAC)	promoters/global/ORFs

Dep1		Rpd3L	promoters/global

Rco1		Rpd3S	ORF

Htz1	H2A.Z	Histone variant	-

Rif1	-	Rap1 interacting factor	-

* KMT = histone lysine methyltransferase, KAT = histone lysine acetyltransferase, HDAC = histone deacetylase.

## Results

### Suppressor analysis of the silencing defect in strains lacking Dot1

**Figure 1:**
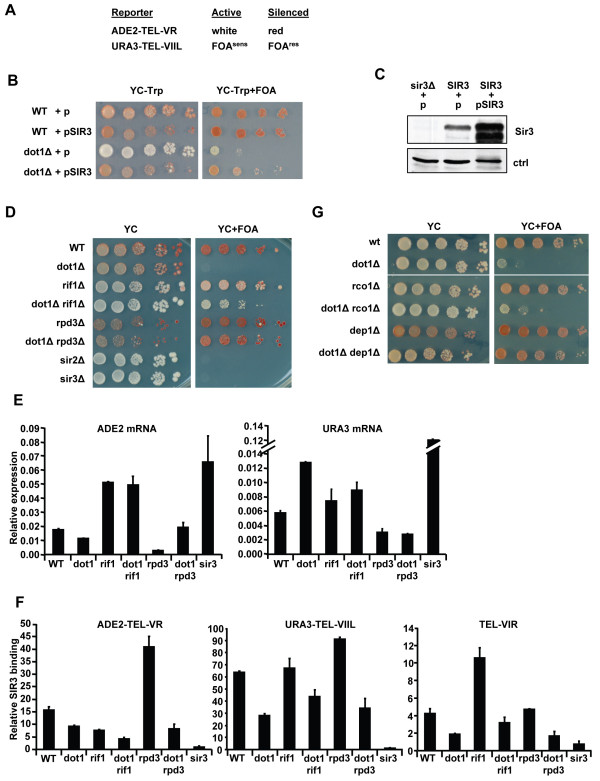
**Suppression of the silencing defects of *dot1Δ *strains**. (A) Reporter genes used for telomeric silencing. Cells in which the *ADE2 *gene is silenced accumulate a red pigment whereas cells that express *ADE2 *are white. Cells in which *URA3 *is silenced are resistant to 5-FOA, whereas cells in which *URA3 *is expressed convert 5-FOA into a toxic product and are sensitive to 5-FOA. (B) Wild-type (WT) and *dot1Δ *strains were transformed with empty vector (p) or a Sir3 overexpression plasmid (pSir3) and were spotted in 10-fold dilution series on media (YC) with and without 5-FOA. (C) Immunoblot analysis of Sir3 expression in *sir3Δ *and WT cells, and cells containing the Sir3 overexpression plasmid. Ctrl indicates a non-specific band recognized by the Sir3 antibody that was used as a loading control. (D) Telomeric silencing in WT and *dot1Δ *strains lacking *RIF1 *or *RPD3*; *sir2Δ and sir3Δ *strains are shown as no-silencing controls (E) mRNA expression levels of *ADE2 *and *URA3 *relative to *ACT1 *were determined by RT-qPCR. mRNA was isolated and quantified in duplicate with the difference as the standard error. (F) Sir3 binding at *ADE2-TEL-VR*, *URA3-TEL-VIIL *and 3500 bp from telomere VIR (VIR3500) relative to binding at control locus *ACT1 *was determined by ChIP combined with real-time qPCR. Each clone was analyzed in duplicate with the difference as the standard error. (G) Silencing in strains lacking *DEP1 *(Rpd3L complex) or *RCO1 *(RPD3S complex).

Previous studies suggest that H3K79 methylation by Dot1 improves targeting of silencing proteins to heterochromatin by preventing promiscuous interactions of Sir3 within euchromatin [5,16,17,28]. To test this hypothesis we investigated three predictions of this model: 1) loss of telomeric silencing in *dot1Δ *cells due to redistribution of the Sir proteins can be overcome by increased expression of Sir3, which is present in limiting amounts, 2) loss of telomeric silencing in *dot1Δ *cells can be suppressed by improving the recruitment of Sir proteins by increasing the strength of the Sir2/3/4-recruiting silencer element, 3) the telomeric silencing defect in *dot1Δ *cells can be suppressed by increased levels of other active marks that affect Sir protein binding or enhanced by decreased levels of these same marks. Our analyses were carried out in a strain carrying two reporter genes: *ADE2 *at the right arm of telomere V (VR) produces a color phenotype and *URA3 *at telomere VIIL provides a sensitive growth phenotype (Figure 1A).

First, Sir3 levels were increased by expression of *SIR3 *from a multi-copy plasmid. Overexpression of Sir3 partially suppressed the silencing defect of the *dot1Δ *strain (Figure 1B–C). Thus, Dot1 is not a critical component of heterochromatin. We note that Sir3 overexpression was not toxic for *dot1Δ *cells (Figure 1B and data not shown) indicating that an increase in Sir3 did not lead to ectopic silencing of essential genes.

**Figure 2:**
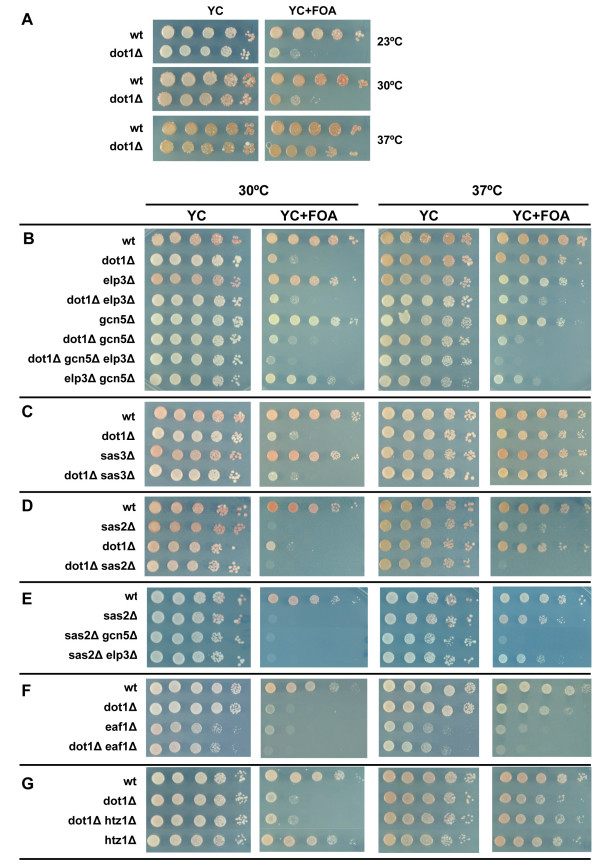
**Multiple euchromatic histone modifiers promote gene silencing by redundant mechanisms**. (A) Analysis of telomeric silencing at 23, 30, and 37°C. (B-G) Single, double and triple mutants of the indicated genes involved in chromatin modification were analyzed as in Figure 1. *dot1Δ *strains have a partial silencing defect and are 5-FOA sensitive at 30°C. Cells were spotted at 37°C to partially restore silencing and identify mutants that enhance the partial silencing defect of *dot1Δ*. Each section represents a different experimental panel.

Second, silencer function of the telomeric repeats was altered. Recruitment of the Sir2/3/4 complex to telomeres is mediated by the telomere-binding protein Rap1 [reviewed in [20]]. Strains lacking the Rap1-interacting factor Rif1 have longer telomeres, which has been suggested to improve recruitment of Sir proteins to the chromosome ends and thereby enhance silencing [29-32]. When *RIF1 *was deleted, silencing of the *URA3 *gene in the *dot1Δ *strain was partially restored (Figure 1D). Using a different approach, we recently showed that Dot1 becomes critical for silencing of the *HML*α locus when the silencer strength at that locus is compromised due to inactivation of Sir1, a silencer-binding protein that facilitates recruitment of the Sir complex to *HML*α [33]. We conclude that the contribution of Dot1 to gene silencing depends on strength of the cis silencer element. Unexpectedly, deletion of *RIF1 *resulted in decreased silencing of the telomeric *ADE2 *gene in wild-type and *dot1*Δ cells in multiple independent clones (Figure 1D and data not shown), whereas a previous study using an *ADE2 *gene at a different telomere showed that deletion of Rif1 made cells more red [32]. Silencing of the *ADE2 *gene and/or color development in strains with no or low expression of *ADE2 *is somewhat variable and can depend on media and growth conditions (e.g. see Figure 2A below and compare WT and *dot1*Δ in Figure 1B with 1D) [34]. This may in part be due to the stochastic nature of *ADE2 *silencing that is observed in yeast colonies [34]. In general, strains lacking Dot1 showed a modest change in color development on complete synthetic media (Figure 1D and see below). To verify whether the changes in colony color and growth on FOA plates were caused by changes in *ADE2 *and *URA3 *expression, respectively, mRNA expression of these genes was determined by reverse-transcriptase combined with quantitative real-time PCR (RT-qPCR). Whereas deletion of Dot1 did not substantially affect *ADE2 *expression under these conditions, deletion of Rif1 caused derepression of the telomeric *ADE2 *gene (Figure 1E). The telomeric *URA3 *gene was derepressed in strains lacking Dot1, and additional deletion of Rif1 partially suppressed the silencing defect. These expression data are in agreement with the color and growth phenotypes of the *rif1Δ *strains (Figure 1D). To verify whether the changes in silencing were caused by changes in Sir protein targeting, binding of Sir3 to the telomeric reporter genes and to a third telomere was determined by chromatin immunoprecipitation (ChIP) combined with qPCR. As expected, Sir3 binding at all three telomeres was reduced in the *dot1*Δ strain (Figure 1F). In the *rif1*Δ and *rif1Δdot1Δ *strains, Sir3 binding was decreased at ADE2-TEL-VR, unaffected or slightly increased at URA3-TEL-VIIL and increased at TEL-VIR (Figure 1F). These results suggest that although deletion of Rif1 can partially suppress the *URA3 *silencing defect of *dot1*Δ cells, the role of Rif1 in silencing is context dependent.

Third, to test whether additional histone modifications are involved in Sir protein targeting, we investigated the consequences of inactivation of the histone deacetylase (HDAC) Rpd3. Acetylation of lysines in the histone tails negatively affects interactions between Sir3 and Sir4 with histones *in vitro *[35,36]. We deleted *RPD3 *because cells lacking Rpd3 activity show increased global levels of histone acetylation in euchromatin [37-43]. Deletion of *RPD3 *enhanced silencing of *ADE2 *in wild-type cells, which is consistent with previous observations [40,44-47], and suppressed the *URA3 *silencing defect of the *dot1*Δ strain (Figure 1D), suggesting that increased acetylation in euchromatin can compensate for the loss of H3K79 methylation. Analysis of *URA3 *mRNA levels confirmed that deletion of Rpd3 improved transcriptional silencing of telomeric *URA3 *in wild-type cells and suppressed the silencing defect of *dot1*Δ cells (Figure 1E). Deletion of Rpd3 also improved silencing of the *ADE2 *gene but did not improve transcriptional silencing of *ADE2 *in cells lacking Dot1 (Figure 1E), despite the similar dark red colony color of *rpd3*Δ and *dot1*Δ*rpd3*Δ cells. We expect that red color development was near saturation in *rpd3*Δ and *dot1*Δ*rpd3*Δ cells due to a combination of partial *ADE2 *silencing and slow growth of cells lacking Rpd3 and that a small reduction in silencing did not result in a color change in these slow growing cells. To investigate how Rpd3 enhanced silencing, binding of Sir3 was examined by ChIP. In wild-type cells, deletion of Rpd3 led to an increase in Sir3 binding at all three telomeres examined (Figure 1F), which supports the idea that global histone acetylation can promote targeting of Sir proteins to heterochromatin. However, in cells lacking Dot1, loss of Rpd3 did not lead to a detectable increase in Sir3 binding (Figure 1F), suggesting that the suppression of the *dot1*Δ silencing defect of telomeric *URA3 *was not caused by restoration of Sir3 binding.

Rpd3 is active in two multi-subunit complexes (see Table 1). The larger Rpd3L complex localizes to promoter regions. The smaller Rpd3S complex is active at transcribed coding regions and is recruited to these regions via the subunits Eaf3 and Rco1, which together bind methylated H3K36, a histone modification co-transcriptionally introduced by Set2 [48-51]. *DEP1 *deletion, which specifically eliminates the Rpd3L complex, phenocopied deletion of *RPD3 *(Figure 1G). In contrast, deletion of *RCO1*, which eliminates the Rpd3S complex, did not affect silencing, showing that the Rpd3L complex and not the Rpd3S promoted gene silencing (Figure 1G). A very recent study identified a third Rpd3 complex, containing Rpd3, Snt2 and Ecm5 [52]. We expect that this complex is not involved in the silencing functions of Rpd3 that we identified here because deletion of *ECM5 *did not affect silencing (data not shown).

Together, the results shown in Figure 1A–G show that Dot1 modulates the strength of gene silencing and that the loss of this modifier can be compensated for by increased Sir3 dosage, strong silencers, and inactivation of Rpd3, a global HDAC.

### Dot1 collaborates with histone acetyltransferases to promote gene silencing

To analyze the genetic relationship between *DOT1 *and other genes involved in euchromatic histone modification, we analyzed silencing of the reporter genes at different temperatures. Growth at high temperature enhances gene silencing in yeast by unknown mechanisms [33,53] and was sufficient to suppress the *dot1*Δ silencing defect (Figure 2A). Perhaps non-specific association of Sir proteins with nucleosomes is decreased at higher temperatures, which improves binding at telomeres. This conditional silencing phenotype provided us with a genetic tool to identify enhancers of the silencing defect in *dot1Δ *strains. Having found that increased acetylation suppressed the *dot1*Δ silencing defect (Figure 1D–E), we examined which HAT might be responsible for the acetylation marks that promote gene silencing. We examined the non-essential HATs Elp3 [54], Gcn5 [55-60], Sas3 [61], and Sas2 [62-64], as well as Eaf1 [65], which encodes the only non-essential subunit of the NuA4 HAT complex (Table 1). Analysis of *dot1Δ *double and triple mutants showed that Elp3, Gcn5, and Sas2 were all required for efficient silencing in *dot1Δ *cells at high temperature (Figure 2B–F). These findings suggest that Gcn5 and Elp3, which have been shown to affect global levels of histone H3 acetylation [41,66-70], and Sas2, which has been shown to be the major H4K16 acetyltransferase [21,62] all promote silencing in parallel to histone H3K79 methylation by Dot1. Analysis of *sas2Δ *in combination with *elp3Δ *or *gcn5Δ *showed that each double mutant had more severe silencing defects than either single mutant, suggesting that the three HATs affect silencing by redundant mechanisms (Figure 2E). Loss of Sas3, which affects bulk histone H3 acetylation when combined with loss of Gcn5 [70,71], did not alter silencing in wild-type or *dot1Δ *cells (Figure 2C), whereas a single deletion of *EAF1 *was sufficient to disrupt telomeric silencing (Figure 2F), even at high temperature (Figure 2F). The histone variant Htz1 (H2A.Z), which is acetylated by NuA4 [72,73] and of which the deposition into chromatin is dependent on Sas2 [74], was not critical for silencing in wild-type or *dot1Δ *strains (Figure 2G).

### Dot1 and Set1 have distinct functions in silencing and cell division

**Figure 3:**
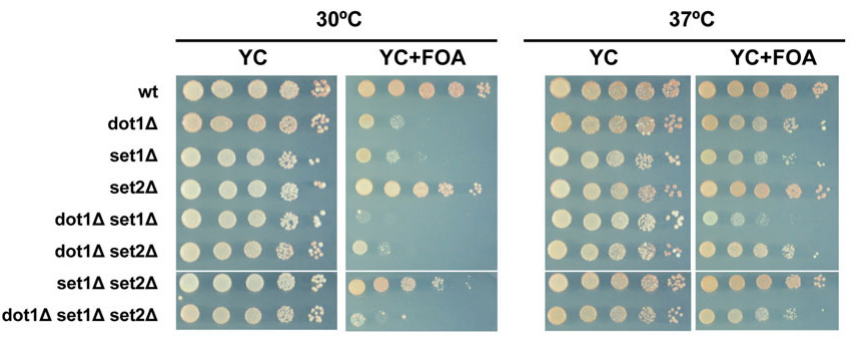
**Histone lysine methyltransferases Dot1, Set1 and Set2 affect silencing by different mechanisms**. Telomeric silencing in strains lacking *DOT1*, *SET1*, and/or *SET2 *was analyzed by growth on media with or without 5-FOA, at 30 and 37°C as described in Figure 1.

**Figure 4:**
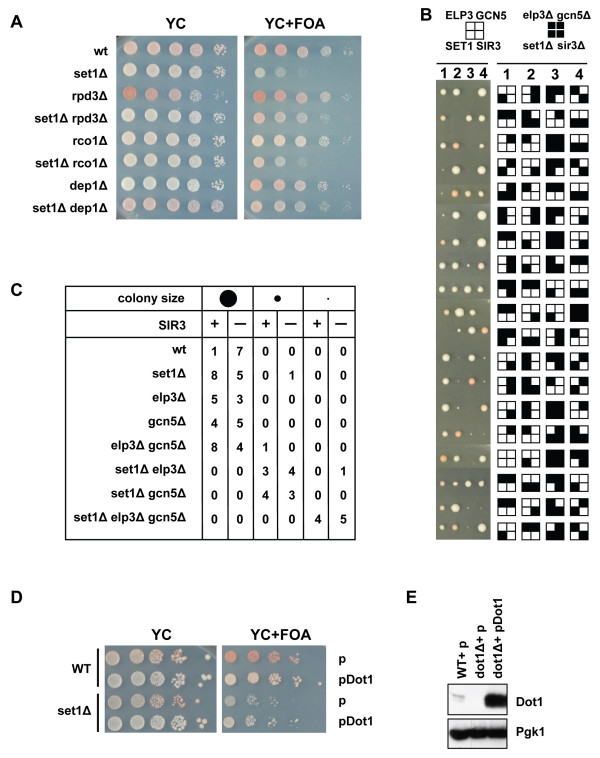
**Silencing and viability of strains lacking Set1 is modulated by histone acetylation**. (A) Deletion of *RPD3 *or *DEP1 *(Rpd3L complex) suppressed the silencing defects of strains lacking *SET1*, whereas deletion of *RCO1 *(Rpd3S complex) had no effect. (B) A diploid strain homozygous for the *ADE2 *and *URA3 *silencing reporters and heterozygous for *SET1/set1::NatMX*, *GCN5/gcn5::HphMX*, *ELP3/elp3::KanMX*, and *SIR3/sir3::HIS3 *was sporulated and spore viability was analyzed by tetrad analysis. Each row indicates the four-spore progeny of one diploid cell. Genotypes and mating type of the individual colonies were determined by replica-plating. Only those tetrads are shown of which the genotype of all four spores could be determined or deduced. The genotype of each colony is indicated by the position of the squares, where white indicates the WT allele and black indicates the mutant allele. (C) Combined deletion of *SET1*, *GCN5*, and *ELP3 *affected cell viability. Colony sizes of panel B (large, small, very small/no colony) were scored for each genotype indicated in *SIR3 *and *sir3Δ *backgrounds. (D) Wild type and *set1Δ *strains were transformed with an empty multi-copy plasmid (p) or a multi-copy plasmid carrying a genomic copy of Dot1 (pDot1) to examine the effect of intermediate levels of Dot1 overexpression on telomeric silencing. (E) Protein levels of Dot1 expressed from its endogenous locus and from the multi-copy plasmid were examined by immunoblot analysis. Pgk1 was used as a loading control and a *dot1Δ *strain was used as a negative control.

Having found that histone H3K79 methylation and various histone H3 and H4 acetylation events influence heterochromatin by distinct mechanisms, we next investigated the relationship between Dot1 and the H3K4 methyltransferase Set1 and the H3K36 methyltransferase Set2. These enzymes are the only known histone lysine methyltransferases in yeast. Strains lacking Dot1, Set1, or Set2 have no detectable methylation of H3K79, H3K4, and H3K36, respectively. Previous studies showed that histone H3K4 methylation by Set1 and H3K79 methylation by Dot1 may affect silencing by a similar mechanism [7,27]. Indeed, overexpression of Sir3 has been shown to rescue telomeric silencing defects of a *set1Δ *strain [27]. Using our silencing reporters, we found that a *dot1Δ set1Δ *double knock-out strain has more severe silencing defects than either single knock-out strain (Figure 3), showing that they work through different mechanisms. *SET2 *deletion did not affect silencing of the two reporter genes in wild-type or *dot1Δ *cells (Figure 3). However, deletion of *SET2 *partially suppressed the silencing defect caused by deletion of *SET1 *(Figure 3), suggesting that some aspect of gene silencing is negatively regulated by the presence of Set2. Methylation of H3K36 by Set2 has been shown to lead to recruitment of the Rpd3S complex to coding regions [48-51]. The negative role of Set2 in gene silencing in *set1Δ *cells (Figure 3) was not mediated by Rpd3S since inactivation of the Rpd3S complex by deletion of *RCO1 *did not improve silencing in a *set1Δ *strain (Figure 4A). Deletion of *RPD3 *or deletion of *DEP1*, which specifically inactivates the Rpd3L complex, did suppress the silencing defects (Figure 4A), indicating that Set1 modulates gene silencing by a mechanism that acts in parallel with Dot1 as well as histone acetylation. To verify this idea, we deleted *GCN5 *and *ELP3 *in the *set1Δ *strain to determine whether reduced acetylation would enhance the silencing defect caused by loss of Set1 function, as we found for Dot1. We noticed that strains lacking Set1 and either one of the two HATs had severe growth defects, especially at 37°C (data not shown). In certain yeast mutants, growth defects are mediated by the Sir complex, presumably because of ectopic Sir-mediated silencing of genes required for cell growth [23,75,76]. These growth defects can be alleviated by deletion of one of the *SIR2/3/4 *genes. To determine whether the growth defects in *set1Δ gcn5 *and *set1Δ elp3Δ *strains were mediated by the Sir complex a diploid strain heterozygous for *SET1*, *GCN5*, *ELP3*, and *SIR3 *was sporulated to generate isogenic single, double, and triple histone modifier knock-out cells that were either silencing proficient (*SIR3*) or deficient (*sir3Δ*) (Figure 4B–C). Tetrad analysis confirmed that haploid spores lacking *SET1 *require *GCN5 *and *ELP3 *for normal growth. Cells lacking all three genes were extremely sick or did not grow at all, indicating that Set1, Gcn5, and Elp3 promote cell division by different mechanisms (Figure 4B–C). Deletion of *SIR3 *did not alleviate any of the growth defects of *set1Δ, elp3Δ *and *gcn5Δ *double and triple knockout strains (Figure 4B–C), which shows that the reduced fitness was not caused by ectopic silencing by the Sir complex.

Since the *set1Δ *silencing defect could be suppressed by increased histone acetylation by inactivation of Rpd3, we asked whether increased activity of Dot1 could also improve silencing and suppress the *set1Δ *silencing defect. To test this, wild type and *set1Δ *strains were transformed with a *DOT1 *multi-copy plasmid (Figure 4D). Under these conditions of Dot1 overexpression (Figure 4E) silencing of the telomeric *URA3 *gene was unaffected in wild-type strains and not or only slightly improved in *set1Δ *strains (Figure 4D). We conclude that the endogenous levels of H3K79 methylation are not limiting for silencing.

### Sir2 and Sir3 expression in strains with silencing phenotypes

**Figure 5:**
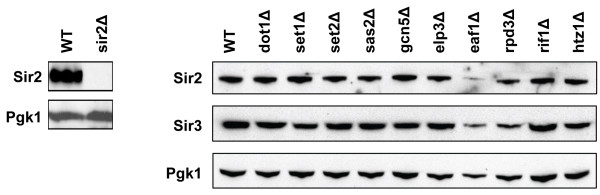
**Expression of Sir2 and Sir3 in strains with altered silencing properties**. Immunoblot analysis of whole-cell protein extracts using antibodies against Sir2 and Sir3. A Pgk1 antibody was used as a loading control. The specificity of the Sir2 antibody is shown in the left panel. The specificity of the Sir3 antibody was shown previously [33] and is shown in Figure 1.

Our results show that heterochromatin function in budding yeast is positively affected by several euchromatic histone modifications and strong silencers. To exclude that phenotypes in the chromatin mutants studied here were caused by altered Sir protein levels we examined the expression of Sir2 and Sir3 by immunoblot analysis. We found that deletion of most of the chromatin modifiers investigated in this study did not affect expression of Sir2 or Sir3 (Figure 5). The immunoblots indicate that expression of Sir2 and Sir3 was reduced in the *eaf1Δ *strain, which might explain why the silencing defect in this strain could not be suppressed by growth at high temperature (Figure 2F) and why Eaf1 affects gene silencing while no changes have been observed in global levels of histone H4 acetylation [65].

## Discussion

**Figure 6:**
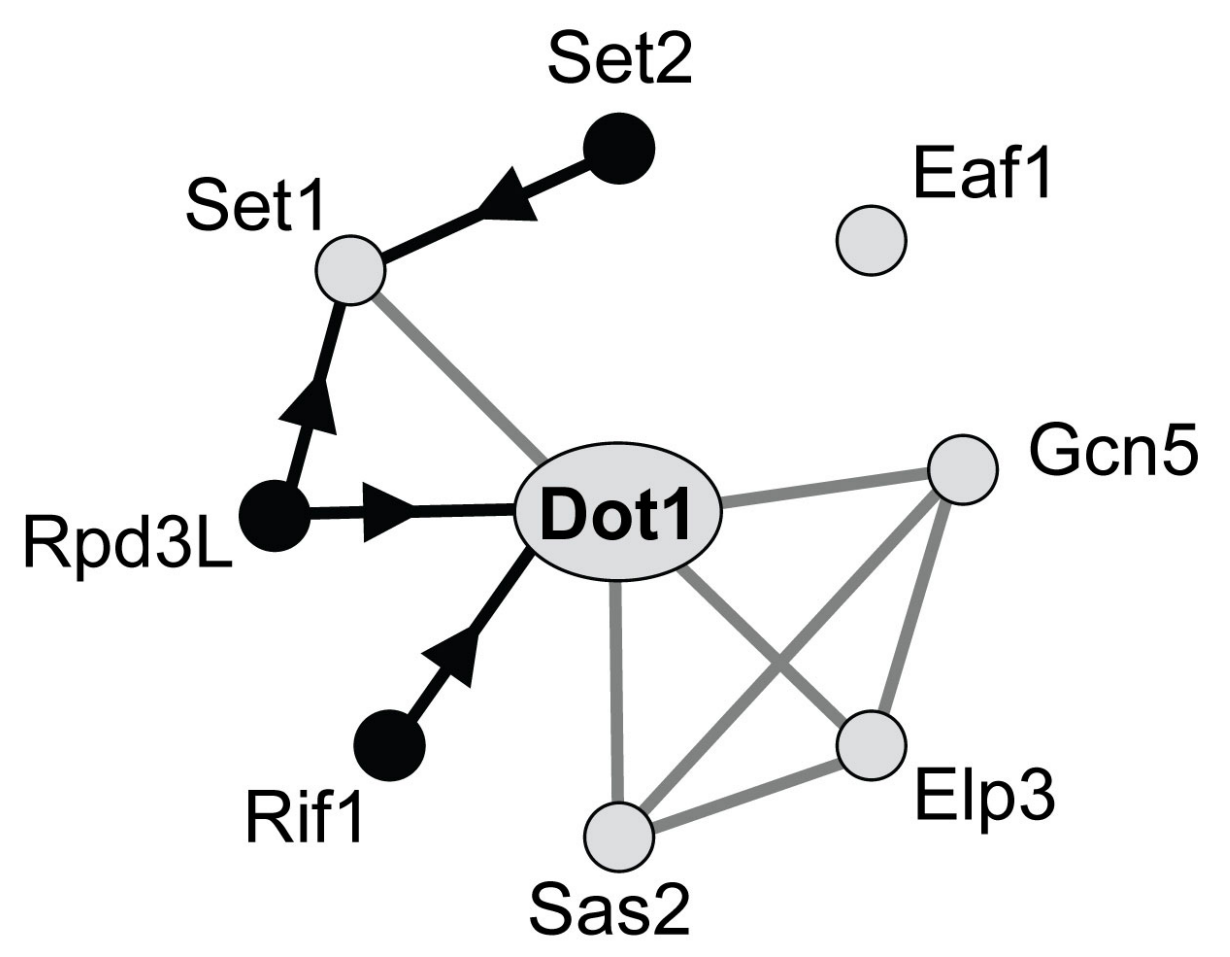
**Summary of genetic relationships identified in this study**. Genetic interactions between Dot1 and other histone modifiers. Grey nodes indicate positive regulators of silencing and black nodes indicate negative regulators of silencing. Grey lines indicate phenotypic enhancement; black lines indicate phenotypic suppression; arrows indicate the directions of the interactions. No silencing phenotypes were observed for *SAS3*, *HTZ1*, and *RCO1*.

We investigated the role of Dot1 in gene silencing by looking for suppressors and enhancers of silencing defects of a *dot1Δ *strain. Our results show that Dot1 is not essential for gene silencing but modulates the efficiency of heterochromatin formation. Silencing in *dot1Δ *strains could be restored by conditions that improve recruitment of the Sir complex: increased Sir3 dosage and stronger silencers. These properties of the *dot1Δ *strains are compatible with a role for Dot1 in enhancing targeting of Sir proteins to regions of heterochromatin. Silencing in wild-type and *dot1Δ *strains was dependent on the activity of other histone modifiers; mutations that increase genome-wide histone acetylation enhanced silencing whereas several mutations that reduce histone acetylation or H3K4 methylation reduced silencing. Therefore, H3K79 methylation and other marks of euchromatin positively influence heterochromatin function by different mechanisms (Figure 6). The redundancy of these pro-silencing functions may reflect differences in genomic locations of the various histone marks. For example, Set2 mono-, di-, and tri-methylates H3K36 mainly in coding regions, Set1 generates mono-, di-, and trimethylated H3K4 preferentially in coding sequence, 5' coding sequence, and promoter regions, respectively, and Dot1 mono-, di-, and tri-methylates H3K79 at similar levels throughout the genome. The redundancy may also reflect the fact that the different marks are located on different areas of the nucleosome and thereby affect multiple contacts between the Sir complex and nucleosome arrays. H3K4, H3K36 and most of the acetylated lysines are located on the tails of histone H3 and H4, while H3K79 is located on the nucleosome core surface and might interact with the tail of histone H4. *In vitro *studies have shown that Sir3 can interact with the histone tails as well as with the nucleosome core and that methylation of H3K4 and H3K79 as well as acetylation of the tail of H3 or H4 can affect Sir-nucleosome interactions. Our genetic studies suggest that binding of the Sir complex to nucleosomes is not affected by post-translational modifications in a combinatorial manner by a specific histone code. Rather, many modifications all seem to make independent contributions to promoting gene silencing. We note that it cannot be excluded at this point that the histone modifying enzymes investigated in this study affect gene silencing by post-translational modification of non-histone proteins or by indirect effects on other histone modifications. For example, Gcn5 acetylates not only histone tails but also other chromatin-associated factors such as Rsc4, a subunit of the RSC nucleosome remodeling complex [68,77]. In addition, Set1 has been shown to influence histone acetylation [78] and to methylate the kinetochore protein Dam1 [79]. Since Gcn5 is also involved in kinetochore function [80], it is perhaps loss of these functions that caused fitness problems in *set1Δgcn5Δ *(and *set1Δelp3Δ*) strains (Figure 4).

**Figure 7:**
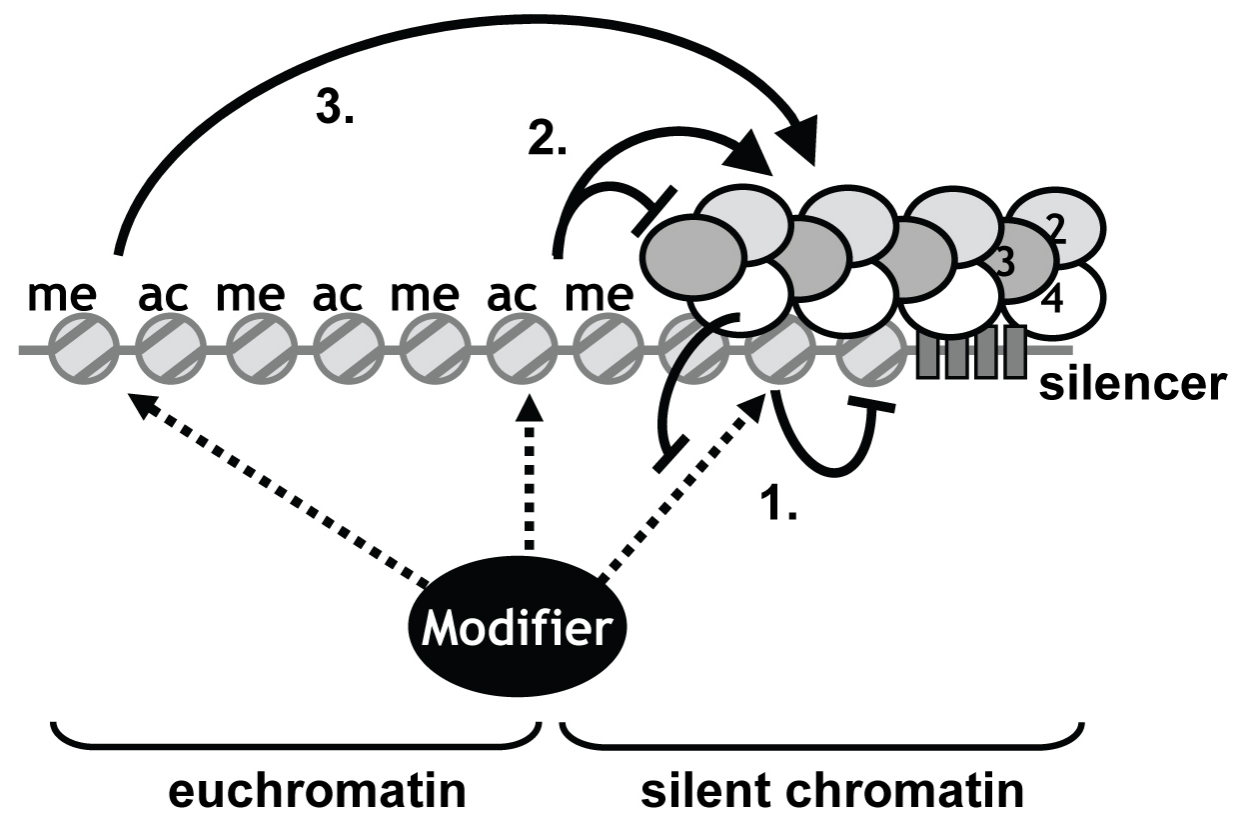
**A competition model for positive and negative roles of euchromatic histone modifications in heterochromatin formation**. Euchromatic histone modifications can have positive roles (arrows) and negative roles (blunt arrows) in heterochromatin formation. Competition between euchromatic histone modifiers and heterochromatin proteins for interactions with nucleosomes can occur at three locations and can have different outcomes (see text). 1) Competition within heterochromatin regions creates a semi-stable epigenetic state. 2) Competition at the interface between euchromatin and heterochromatin prevents local spreading of the Sir complex, thereby on the one hand avoiding ectopic silencing of regions adjacent to heterochromatin and on the other hand ensuring availability of limiting silencing proteins for the endogenous heterochromatic regions. 3) Competition throughout euchromatin prevents non-specific binding of the Sir2/3/4 complex to bulk chromatin, thereby enhancing targeting of Sir proteins to endogenous heterochromatic regions to ensure sufficient spreading of the Sir complex. By these mechanisms, the function of a euchromatic histone modification in gene silencing depends on the relative contribution that it makes to each of these mechanisms and to what extend the negative and positive functions counteract each other.

Heterochromatin in yeast is characterized by the absence of post-translational modifications of histone proteins and biochemical and genetic studies indicate that binding of Sir proteins to nucleosomes is negatively affected by histone methylation and acetylation. Therefore, heterochromatin formation and spreading seems to be determined by a competition between binding of the Sir complex and action of euchromatic histone modifying enzymes. Competition between histone modifiers and the Sir proteins can in principle affect heterochromatin at three distinct genomic or chromatin locations (Figure 7). First, histone modifiers can compete with Sir proteins within heterochromatin domains. By doing so they are expected to destabilize heterochromatin domains. Indeed, when Sas2 or Dot1 are overexpressed and lead to increased global histone acetylation and methylation, respectively, Sir protein binding and silencing at telomeres is reduced [5,17,28,81]. Furthermore, the presence of Sas2 and Dot1 in yeast cells delays the onset of silencing at a previously active locus [82]. Second, modifying enzymes can deposit an anti-silencing mark at the interface between euchromatin and heterochromatin and thereby form a boundary. By this mechanism, loss of a histone modifying enzyme is expected to lead to increased spreading of heterochromatin into adjacent regions. When excessive spreading occurs of the limiting Sir proteins this may be accompanied by reduced Sir protein binding and impaired silencing of the distal wild-type silenced loci, as has been observed for strains lacking *SAS2*, *BDF1 *or *GCN5+ELP3 *[21,22,75,83]. Third, histone modifications throughout euchromatin can prevent non-specific binding of Sir proteins, which increases the availability of the limiting Sir proteins for heterochromatic regions [19]. By this model, loss of a modifying enzyme is expected to reduce targeting of Sir proteins to heterochromatic areas as well as their flanking regions, as has been described for *DOT1 *and *SET1*. In cells lacking Dot1 or Set1, Sir proteins do not spread excessively but become redistributed throughout the genome [5,25,26]. Also by this model, higher levels of a global and limiting histone modification are expected to improve targeting, silencing, and ectopic spreading of Sir proteins, in which case the degree of spreading will depend on how the increased mark affects competition within heterochromatin and at the boundary.

Our results and previous studies show that multiple euchromatic histone modifiers influence telomeric silencing and here we show that many of them seem to act by non-overlapping mechanisms. How might they affect gene silencing? Deletion of a histone modifier generally does not enhance silencing (e.g. Figures 2, 3 and 4). Therefore, they do not seem to weaken silencing by local competition with heterochromatin formation. Several factors might determine the importance of local competition. For example, euchromatic modifiers might not have access to the nucleosomal substrates in heterochromatin [17,84], local disruption of silencing by histone modifiers might be counteracted by (indirect) positive silencing effects, or post-translational modification in heterochromatin might be counteracted by histone-demodifying activities such as the HDAC activity of Sir2 and the H2B deubiquitinating activity of UBP10, which is recruited to heterochromatin by Sir4 and negatively affects methylation by Dot1 and Set1 [85,86]. We expect that Dot1, Set1, Gcn5, Elp3, and Sas2, which we investigated here, act at least in part by long-distance targeting effects because they deposit abundant histone modifications throughout the euchromatic genome and loss of the modifying enzymes leads to reduced silencing. Similarly, based on the phenotypes of *rpd3Δ *cells i.e. more spreading into flanking regions (Figure 1 and [31]) as well as increased silencing within wild-type heterochromatin regions (Figures 1 and 4), we propose that deletion of *RPD3 *leads to improved targeting of silencing proteins to heterochromatin domains, which can overrule the local boundary and lead to ectopic spreading. Set2 and Htz1 might act by a similar mechanism. Strains lacking Set2 show increased spreading [87] as well as increased silencing within existing heterochromatin regions in a *set1Δ *strain (Figure 4) and strains lacking Htz1 show increased spreading [24] without loss of silencing at wild-type heterochromatin loci (Figure 2). These silencing phenotypes suggest that Set2, Htz1, and Rpd3 might not act as local boundary factors, as has been proposed previously [23,24,31,87], but instead affect the degree of spreading from a distance by reducing the targeting efficiency of Sir proteins to heterochromatin. Thus, these factors might set heterochromatin boundaries by weakening Sir-protein targeting to heterochromatin domains.

The mechanism by which Rpd3 weakens silencing is still unclear, however. Whereas increased gene silencing in *rpd3Δ *cells (Figure 1D+E) was accompanied by increased Sir3 binding (Figure 1F), deletion of Rpd3 in *dot1Δ *cells restored *URA3 *silencing without any detectable changes in Sir3 binding (Figure 1D–F). Although Sir3 is the major Sir protein that can spread along the chromosome over long distances, these results suggest that factors other than Sir3 restored silencing in *rpd3Δdot1Δ *cells. Whether Rpd3 affects binding of one of the other Sir proteins remains to be determined. Changes in silencing without detectable changes in Sir protein binding have been described previously, however [88]. Therefore, it is also possible that Rpd3 might affect the activity of the Sir proteins, such as the deacetylase activity of Sir2, or targeting of repressor proteins that can act as heterochromatin factors but are normally not found in Sir-occupied heterochromatin domains, such as Sum1, Hst1, or Hda1 [89-94]. Interestingly, our results also indicate that the effect of Rpd3 on Sir3 targeting to telomeric heterochromatin requires the presence of Dot1. This suggests that targeting of Sir proteins to heterochromatin by increased histone acetylation might involve simultaneous recognition of methylated H3K79. The redundant roles of Dot1 and the histone H3 HATs Gcn5 and Elp3 in promoting gene silencing indicates that histone H3 acetylation and H3K79 methylation involve non-linked mechanisms. To fully understand the connection between Dot1 and Rpd3, it will be important to determine which of the many target lysines of Rpd3 affect Sir3 targeting in a Dot1-dependent manner and whether Rpd3 and Dot1 affect each others activity.

## Conclusion

Targeting of the Sir complex to regions of heterochromatin in yeast is positively modulated by a range of euchromatic factors indicating that euchromatin and heterochromatin are interdependent. Our results and previous studies suggest that histone modifiers can compete with heterochromatin proteins at different locations and thereby make positive as well as negative contributions to heterochromatin formation. We expect that similar rules apply to histone modifications in higher eukaryotes, which might help to explain the paradoxical role of Dot1 in gene activation and repression in flies and mammals [8,9,11,95-98].

## Methods

Yeast strains are described in Table 2. Gene knock-outs were made by replacing the coding sequences of the respective genes by homologous recombination with PCR products of plasmids pRS400 (KanMX), pRS40NatMX, pRS40HygMX, or pRS303 (*HIS3*) [33,99]. Most gene deletions were generated as heterozygous diploids. Haploids were subsequently obtained by sporulation. For spore analysis of the *elp3Δgcn5Δset1Δsir3Δ *heterozygous diploid (NKI1121) genotypes of the four spores of each tetrad were determined by drug resistance, histidine prototrophy, and mating to tester strains PT1a and PT2α [34]. Genotypes that could not be determined directly (due to synthetic lethality or due to loss of mating type caused by the absence of Sir3) were deduced from the segregation of the markers in the remaining spores. Only tetrads of which the genotypes of all four spores could be unambiguously assigned were included for further analysis. Yeast media were prepared and silencing assays were performed as previously described [28,34] and repeated at least two times. Silencing assays were performed with cells that had been pre-grown at the appropriate temperature for at least one day. Plasmids pRS424 (2 μ-*TRP1*) and pHR67-23 containing a *SIR3 *genomic region in pRS424 were described previously [33,99].

**Table 2:** Strains used in this study

**Strain**	**Relevant genotype (all strains are isogenic to UCC7366)**
UCC7366	MAT**a **lys2Δ0 trp1Δ63 his3Δ200 ade2Δ::hisG ura3Δ0 leu2Δ0 met15Δ0 ADE2-TEL-VR URA3-TEL-VIIL

NK1121	MAT**a**/α ELP3/elp3Δ::KanMX GCN5/gcn5Δ::HphMX SET1/set1Δ::KanMX SIR3/sir3::HIS3

UCC7356	dot1Δ::NatMX

UCC7367	set1Δ::KanMX

NKI5154	set1Δ::NatMX

UCC7368	set2Δ::HphMX

NKI5092	gcn5Δ::HphMX

NKI5090	elp3Δ::KanMX

NKI5183	sas2Δ::NatMX

NKI5185	sas2Δ::HphMX

NKI5142	sas3Δ::KanMX

NKI5048	htz1Δ::HphMX

NKI5372	eaf1Δ::HphMX

NKI1001	rif1Δ::HphMX

NKI5061	rpd3Δ::HphMX

NKI5152	dep1Δ::HphMX

NKI5148	rco1Δ::HphMX

UCC7359	dot1Δ::NatMX set1Δ::KanMX

UCC7357	dot1Δ::NatMX set2Δ::HphMX

UCC7360	set1Δ::KanMX set2Δ::HphMX

UCC7361	dot1Δ::NatMX set1Δ::KanMX set2Δ::HphMX

NKI5088	dot1Δ::NatMX gcn5Δ::HphMX

NKI5086	dot1Δ::NatMX elp3Δ::KanMX

NKI5083	gcn5Δ::HphMX elp3Δ::KanMX

NKI5082	dot1Δ::NatMX gcn5Δ::HphMX elp3Δ::KanMX

NKI5226	dot1Δ::NatMX sas2Δ::HphMX

NKI5144	dot1Δ::NatMX sas3Δ::KanMX

NKI5046	dot1Δ::NatMX htz1Δ::HphMX

NKI5374	dot1Δ::NatMX eaf1Δ::HphMX

NKI1004	dot1Δ::NatMX rif1Δ::HphMX

NKI5059	dot1Δ::NatMX rpd3Δ::HphMX

NKI5152	dot1Δ::NatMX dep1Δ::HphMX

NKI5146	dot1Δ::NatMX rco1Δ::HphMX

NKI5224	set1Δ::NatMX rpd3Δ::HphMX

NKI5322	set1Δ::NatMX dep1Δ::HphMX

NKI5323	set1Δ::NatMX rco1Δ::HphMX

NKI5212	sas2Δ::NatMX gcn5Δ::HphMX

NKI5210	sas2Δ::NatMX elp3Δ::KanMX

### Immunoblots

Whole-cell extracts were obtained from approximately 5 × 10^7 ^cells by the classical glass beads breakage method using 200 μl of glass beads and SUMEB (1% SDS, 8 M Urea, 10 mM MOPS pH 6.8, 10 mM EDTA, 0.01% bromophenol blue, 1 mM DTT) [100] complemented with PMSF (1 mM), benzamidine (5 mM), pepstatin (1 μg/ml), leupeptin (1 μg/ml) and DTT (1 μM). Primary antibody incubations were performed in Tris-buffered saline-Tween with 2% dry milk. Antibodies used were Sir3 [33], Dot1 [28], Sir2 (Santa Cruz, Sc-6666), and Pgk1 (Invitrogen, A-6457).

### Reverse-transcription

Total yeast RNA was prepared from 5 × 10^7 ^cells of each of the indicated growth condition using the RNeasy kit (Qiagen) according to the manufacturer's protocol. RNA samples were treated with RNase free DNAse (Qiagen), and cDNA was made by using Super-Script II reverse transcriptase (Invitrogen).

### Chromatin immunoprecipitation

ChIP was performed as described previously [101]. Briefly, the chromatin was sheared using a bioruptor (Diagenode) for 6 minutes with 30 seconds intervals at high. The obtained fragments have an average size of 500 bp, as determined on a 2% TAE gel stained with ethidium bromide and quantified using TINA software. The isolated chromatin of the equivalent of 5 × 10^7 ^cells was immunoprecipitated overnight at 4°C using magnetic Dynabeads (Invitrogen) which were previously incubated with antibody o/n at 4°C.

### Real-time PCR

ChIP DNA and cDNA was quantified by real-time PCR using the SYBR^® ^Green PCR Master Mix (Applied Biosystems) and the ABI PRISM 7500. A ChIP input sample was used to make a standard curve, which was then used to calculate relative IP efficiencies and mRNA expression levels, using the 7500 fast system software. Primers used for qPCR are listed in Table 3.

**Table 3:** qPCR primers used in this study

**RT-PCR Primers**	**Sequence**
ACT1_QforORF	TCGTTCCAATTTACGCTGGTT

ACT1_QrevORF	CGGCCAAATCGATTCTCAA

ADE2_ORF_Qfor	TTGGGTTTTCCATTCGTCTTG

ADE2_ORF_Qrev	CAACGAAGTTACCTCTTCCATCGT

URA3orf_Qfor	GGGCAGACATTACGAATGCA

URA3orf_Qrev	CCTGCTTCAAACCGCTAACAA

	

**ChIP Primers**	**Sequence**

ACT1_Qfor	CTCTTTTTATCTTCCTTTTTTTCCTCTCT

ACT1_Qrev	CGTGAAAAATCTAAAAGCTGATGTAGTAG

ADE2_ORF_Qfor	TTGGGTTTTCCATTCGTCTTG

ADE2_ORF_Qrev	CAACGAAGTTACCTCTTCCATCGT

URA3_Qfor	GGAAGGAGCACAGACTTAGATTGG

URA3_Qrev	CTGTGCAGTTGGGTTAAGAATACTG

VIR3500_Qfor	CCCATGTTTTTCAGTTTATCAATGA

VIR3500_Qrev	CGATGAAGATTGTATGCAAGCAA

## Competing interests

The authors declare that they have no competing interests.

## Authors' contributions

KFV, AWF, and FvL designed the experiments. AWF and FvL made the yeast strains and performed silencing assays. FvL and KFV performed tetrad analyses. KFV performed qRT-PCR and ChIP analyses. IJES and FvL performed immunoblots. FvL conceived the study. KFV, IJES, and FvL wrote the paper. All authors read and approved the final manuscript.
